# Borreliosis and Other Tick-Borne Diseases in the Northern and Southern Hemispheres

**DOI:** 10.3390/microorganisms13112418

**Published:** 2025-10-22

**Authors:** Giusto Trevisan, Serena Bonin, Nestor Oscar Stanchi

**Affiliations:** 1 Department of Medical Sciences, University of Trieste, 34149 Trieste, Italy; sbonin@units.it; 2 Facultad de Ciencias Veterinarias, Universidad Nacional del Chaco Austral, Roque Sáenz Peña 3700, Argentina; nestorstanchi@uncaus.edu.ar

## 1. Editorial

Lyme disease (LD) is an anthropozoonosis caused by the spirochaete *Borrelia burgdorferi* sensu lato (Bbsl), which is transmitted by ticks belonging to the genus - *Ixodes.*

To date, 24 species belonging to the *Borrelia* Lyme Group (LG) have been identified, 9 of which are pathogenic to humans. Their identification has been achieved through phylogenetic analysis [1], whole-genome sequencing and/or multi-locus sequence typing (MLST) based on eight genes [2].

LD continues to expand its geographic and epidemiological incidence beyond North America and Eurasia. Its presence has also been documented in Africa [3] and South America [4].

The articles presented in this issue reflect the complexity of LD in diverse contexts, from Europe, North and Latin America to urban environments, where the risk has often been underestimated. Collectively, the papers illustrate the urgent need for integrated approaches to surveillance, diagnosis, and prevention [5].

Data from Poland [6] and the Netherlands [7] reveal high incidence rates, often underestimated due to gaps in surveillance systems. Cohort studies in Slovenia and Italy provide detailed clinical and microbiological insights, with a prevalence of *Borrelia afzelii* [8].

The identification of pediatric LD cases is particularly challenging due to variations in its incidence, the scarcity of notifications and deviations from recommended practices, which remain significant [9].

The contributions collected here highlight the diverse dimensions of Lyme disease. Retrospective clinical analyses from Argentina highlight the ongoing debate about the presence of *Borrelia* in South America. Reports from Brazil describe the characteristic Baggio-Yoshinari syndrome (BYS), which reflects the unique ecological and biological conditions of South America [10].

Experimental research in the United States is exploring novel therapeutic targets such as inhibition of IMPDH/GuaB, which is required for Bbsl replication in mammalian hosts and therefore the use of GuaB inhibitors could be considered for the future development of new drugs for the treatment of LD [11].

Despite regional differences, common challenges emerge. Diagnosis is often clinical, due to the presence of erythema migrans (EM), which represents the first stage of the disease (localized early stage). Diagnostic features are characterized by a circular erythema, which appears around the tick bite not immediately, but after an incubation period of 4–30 days, with a diameter of at least 5 cm. If left untreated, it enlarges and can reach a diameter of even more than 20–30 cm, sometimes taking on the typical bull’s-eye appearance [12].

The presence of EM allows antibiotic treatment to be started immediately, especially considering that serology may still be negative at this stage. EM is present in 75% of cases and is most often localized to the lower limbs, trunk, and upper limbs, while localization to the face is more frequent in children; in these cases, treatment should be started even more immediately, due to the increased risk of developing facial manifestations such as facial paralysis or involvement of the temporomandibular joint, with masticatory disorders [13]. In a quarter of cases, where EM has not been identified, the diagnosis remains difficult: the presence of clinical manifestations such as conjunctivitis [14], migrating myoarticular pain [15], headache or other neurological symptoms [16], arrhythmia [17], requires anti-Borrelia antibodies detection, which is essential for diagnosis at this stage.

These tests are currently reliable, with the inclusion of recombinant antigens [18] and can be performed by ELISA, CLIA, and Western blot, which indicates the positive bands. In North America, serological testing mainly refers to *Borrelia burgdorferi* sensu stricto (Bbss) rarely *B. mayonii* [19], as this species is by far the most common, while it is more complex in Eurasia, as the *Borreliae* species that cause LB are more numerous (*Borrelia garinii*, *B. bavarensis*, *B. afzelii*, *B. valaisiana*, Bbss, *B. lusitaniae*, *B. bissettii*), and serology with chimeric antigens has also been proposed [20].

New tests are currently under development for the diagnosis of LD. Among these, a PCR-based method targeting the Bbsl prophage gene *TerL* has shown higher sensitivity compared to conventional bacterial DNA detection [21], although its applicability appears limited to Bbss [22]. Also a proteomic approach has been reported to identify Bbsl antigens in body fluids, with potential future applications in detecting microbiologically active forms of LD [23].

Surveillance remains fragmented, resulting in underestimation even in highly endemic regions. Furthermore, climate change, environmental modifications, and migratory birds are facilitating the expansion of ticks and *Borreliae* Lyme Group into new territories [24], raising important questions for countries previously considered non-endemic, particularly Asia, South America and Africa.

The Portuguese experience in companion animals exemplifies how veterinary data can serve as an early warning system for human health risks. Analysis of pathogens present in ticks (*Rhipicephalus sanguineus*, *Ixodes ricinus*, *Hyalomma*, and *Dermacentor* spp.) has documented the presence of *Rickettsia*, *Bartonella*, TBEv, and *Crimean-Congo Hemorrhagic Fever Virus* (CCHFV), and the resulting underestimated zoonotic risks in companion animals, as part of an early warning system within a One Health surveillance approach [25].

Similarly, ecological and environmental surveillance, as documented by studies carried out on *Ixodes* ticks in Estonia, caught in urban recreational areas showing the presence of Bbsl, and also of other infectious agents: *Rickettsia* spp., *Noehrlichia mikurensis*, *Borrelia miyamotoi* (agent of Hard Tick-Borne Relapsing Fever), *Anaplasma phagocytophilum* and TBEv, demonstrates that urban areas may present risks comparable to natural endemic areas [26].

Lessons learned from other zoonotic diseases, such as leptospirosis [27], further illustrate the value of integrated approaches to prevention and control. They highlight the often underestimated zoonotic risks in urban green spaces.

Future efforts must focus on strengthening international scientific collaboration, ensuring the validation of diagnostic tools in regions where LB and other tick-borne diseases (TBDs) remain controversial, and improving the training of primary care health workers [28]. Equally important is the training of new generations of researchers trained to work interdisciplinarily, bridging the fields of human, veterinary, and environmental health medicine.

LD is present and endemic primarily in the Northern Hemisphere but is slowly spreading also to areas toward the equator and even into the Southern Hemisphere. This issue contributes to improving knowledge on the spread of LB, particularly in South America [29].

## 2. Conclusions

Lyme disease is not a challenge limited to specific countries or continents, but a growing global health concern. The papers included in this issue demonstrate the importance of collaborative research and shared knowledge.

Only through joint efforts can we hope to improve diagnosis, enhance surveillance, and mitigate the impact of Lyme borreliosis worldwide.

## Data Availability

No new data were created or analyzed in this study. Data sharing is not applicable to this article.

## Abbreviations

The following abbreviations are used in this manuscript:

LD	Lyme Disease
EM	Erythema migrans
Bbls	Borrelia burgdorferi sensu lato
Bbss	Borrelia burgdorferi sensu stricto
